# Which carbon footprint for my ICU? Benchmark, hot spots and perspectives

**DOI:** 10.1186/s13613-025-01445-z

**Published:** 2025-03-20

**Authors:** Pierre Bardoult, Elodie Cadic, Olivier Brichory, Véronique Marie, Caroline Rouxel, Christophe Millet, Magalie Daudin, Elodie Peguet, Nicolas Massart

**Affiliations:** 1 Service de Réanimation, CH de St BRIEUC, 10, Rue Marcel Proust, 22000 Saint-Brieuc, France; 2 Anesthesia and Intensive Care Department, Univ Rennes, CHU Rennes, Inserm, COSS 1242, F-35000 Rennes, France; 3 Direction des achats et de la logistique, CH de St BRIEUC, 10, Rue Marcel Proust, 22000 Saint-Brieuc, France; 4 Service d’hygiène Hospitalière, CH de St BRIEUC, 10, Rue Marcel Proust, 22000 Saint-Brieuc, France; 5 Service d’Urgence, CH de St BRIEUC, 10, Rue Marcel Proust, 22000 Saint-Brieuc, France; 6 Service de Cardiologie, CH de St BRIEUC, 10, Rue Marcel Proust, 22000 Saint-Brieuc, France; 7 Service de Pharmacie, CH de St BRIEUC, 10, Rue Marcel Proust, 22000 Saint-Brieuc, France

**Keywords:** Life cycle assessment, Critical care, Global warming

## Abstract

**Background:**

The purpose of this study was to identify the main greenhouse gas (GHG) emitting activities or products among the medical devices (MD) and medicines used in a polyvalent Intensive Care Unit (ICU).

**Methods:**

A pragmatic eco-audit was conducted in a 21-beds polyvalent ICU, in Saint-Brieuc, Bretagne, France. It consisted of estimating GHG emissions of products or activities, considering process-based life cycle analysis (LCA), economic input–output analysis (EIO) and hybrid-LCA. Results were expressed as Carbon Dioxide Equivalent (CO_2_*e*) emissions per patient-day considering each medication and MD (including personal protective equipment).

**Results:**

With remaining uncertainty, GHG emissions were estimated at 61.1 kgCO_2_*e* per patient-day. Two hundred and two individual MD were used per patient-day, equivalent to 5.1 kgCO_2_*e* per patient-day (process-based LCA). Gloves accounted for the main part of kgCO_2_*e* emissions (representing 1.8 kgCO_2_*e* per patient-day). Then, syringes (1.1 kgCO_2_*e* per patient-day), perfusion tubings (1.0 per patient-day) and gauze pads (0.4 kgCO_2_*e* per patient-day) were the most important sources of MD related GHG emissions. Forty-seven individual medicines were used per patient-day. Most consumed medications were sterile water for injection, propofol, and sodium chlorure. The GHG emissions of medications were estimated with EIO-LCA at 21.5 kgCO_2_*e* per patient-day, mostly due to injectable medicines (15.3 kgCO_2_*e* per patient-day).

**Conclusion:**

Upcoming studies focusing on actions on these particular hot spots would be of interest in order to significantly decrease GHG emissions but also to increase resilience of critical care.

**Supplementary Information:**

The online version contains supplementary material available at 10.1186/s13613-025-01445-z.

## Take home messages:

- GHG emissions related to medical devices were estimated around 5.1 kgCO_2_*e* per patient-day.
- Main sources of GHG emissions related to medical devices in our ICU were gloves and devices related to pharmaceutical IV administration (especially syringes, perfusion tubing and gauze pads).
- A lack of data on the GHG emissions related to medicine production was observed, drastically affecting estimation of GHG emissions. Using economic input output analysis, GHG emissions related to medications were estimated at 21.5 kgCO_2_*e* per patient-day, mostly due to injectable medicine (15.3 kgCO_2_*e* per patient-day).

## Background

Climate change is the greatest challenge of this century [1], as it is already transforming our society, health and healthcare systems. If adaptation to future change is an issue, reducing our greenhouse gas (GHG) emissions is crucial as they are the driver of global warming.

In France, health sector accounts for 6.6% to 10% of French GHG emissions, with almost 40% coming from the hospital sector [2]. Intensive care units (ICUs) are environmental hot spots because of their high consumption of medicines, medical equipment and energy needed to provide a high level of care. Moreover, most consumed medicines and medical devices (MD) are constituted of synthetic plastics, rubbers and fabrics, that mostly depend on petroleum derivates [3]. Stress regarding resource availability will consistently alter supply chain. Intensivists must evaluate environmental impact of their practices and find out alternatives, in order to reduce carbon footprint and to adapt to future major changes.

To date, few studies focused specifically on ICUs environmental impact. A recent review [4] including 13 studies estimated daily GHG emissions in between 88 and 178 kg of Carbon Dioxide Equivalent (kgCO_2_*e*) per patient-day. However, these results must be interpreted with caution since the majority of studies were conducted in Australian or American ICUs [5– 8]. Energy use, which is mainly based on fossil fuels in these countries, transport and health systems are different and reduce the external validity. Therefore, the results cannot be generalized worldwide and GHG emissions in Europe are required. In addition, most of the studies focused on waste only. Two studies were conducted with Life Cycle Assessments (LCA) [5, 6]. Another study, conducted in the Netherlands by Hunfeld et al., focused on material flows [7], but did not include energy consumption and medicines, which are known to be responsible for a high proportion of GHG emissions in ICUs. These results need to be incorporated into an exhaustive assessment in order to better identify the most effective actions to reduce GHG emissions of critical care.

The purpose of this study was to identify products or activity with the highest global warming impact (referred to as "hot spots") among the MD and medicine used in our 21-bed polyvalent ICU.

## Methods

### Setting

Saint-Brieuc ICU is a 21-bed polyvalent ICU in a 750 acute care beds hospital in Côtes-d’Armor, Bretagne, France, covering a 600, 000 inhabitants’ area.

### Unit measurement

As CO_2_ is the GHG used as a reference to estimate global warming potential, the results were expressed as CO2 equivalent emissions, expressed as kgCO_2_*e*. To illustrate, a French inhabitant produces an average of 6, 900 kgCO_2_*e* per year.

### Eco-audit and life cycle assessment boundaries

The eco-audit consisted in estimating GHG emissions directly related to critical care in our ICU for the year 2022. The functional unit was mean carbon dioxide equivalent (CO_2_*e*) emissions per patient-day (a day of stay for one patient) considering each medical device, including Personal Protective Equipment, and medicine consumed.

We intentionally focused on MD and medicines that were consumed in large amount. References that were used only occasionally (less than 30 units per year for medication and less than 400 units per year for MD) were not assessed. The identified sample of consumption represented almost all MD consumed and a large part of medicine consumption. Noteworthy, the remaining medications used less than 30 times a year represented a large number of references, consumed in highly variable amount each year. It is likely that dedicated action regarding these specific items will require a large amount of work for limited impact.

GHG emissions can be distributed into three scopes: scope 1 or direct emissions (related to energy or fuel combustion) related to facility function, scope 2 or indirect emissions, associated with the purchase of electricity or cooling, and scope 3; representing all other indirect emissions, which include water and waste, commuting and alimentation, medicine, equipment [4].

Different methods of GHG emissions calculations were applied. Process-based and Economic Input–Output (EIO) LCAs were performed for MD and medicines (scope 3).

For exploratory purpose, medical gas consumption (scope 1), energy consumption (scope 1 and 2), patients and caregivers alimentation and caregivers transport (scope 3) were assessed through a hybrid LCA (details regarding methods in supplementary data). GHG emissions that occurred directly inside the study area (refrigerant liquid gas) were out of the scope of the study. GHG emissions related to Saint-Brieuc hospital real estate, support function of the hospital (logistic, human resource, finance, security) and visitors transportation (Scope 3) were out of the scope of the study. However, energy consumption related to laundry and sterilization (electricity and gas) was considered with this estimation (see supplementary data for details regarding energy analysis, unit measurement and inflows/outflows).

### Process-based life cycle assessment

A process-based LCA was performed for MD and medicines, estimating related GHG emissions. LCA is a method for assessment of a service from raw material extraction to waste treatment. It corresponds to the burden imposed on the global warming impact by a product or a service and it accounts for the resource and energy consumed at each stage (input) and the resulting wastes (outputs). Global GHG emissions of each product were considered, including the GHG emissions related to production, packaging, transport and waste elimination.

i) Regarding raw material production, each element was examined to evaluate each single component of the final product. Then, the weight of each component of each product (for example polypropylene) was multiplicated by its corresponding GHG emission factor (0.02 kgCO_2_*e*/kg for polypropylene) to obtain corresponding GHG emissions in the present situation. Datas used for GHG emissions assessment were obtained from the website “Base empreinte” of the “Agence de la transition écologique” (https://base-empreinte.ademe.fr). Importantly, manufacturing step was not considered; as it is variable across countries and manufacturer, but only primary production was considered.
ii) Packaging were analyzed similarly, based on composition of packaging, corresponding weight and GHG emission factor. Sterilization and packaging manufacturing steps could not be evaluated.
iii) Transport was evaluated from the factory to our ICU. It was assumed that for transport from Asia, maritime transport (0.0109 kgCO_2_*e*/ton/km) was applied while from Europe, trucks were used (0.126 kgCO_2_*e*/ton/km for foreign transportation and 0.0799 kgCO_2_*e*/ton/km for French transportation). For maritime transport, a supplementary impact was added for the transport from major European harbor (Rotterdam) to our ICU by trucks.
iv) Waste elimination was estimated using currently used disposal sectorization. It included elimination related to the product but also to its packaging. The weight of each waste was multiplicated by the corresponding GHG emissions factor of the corresponding sector as reported by Base Empreinte corresponding to 943 kgCO_2_*e* per ton of waste for healthcare associated waste (bio-hazardous waste, incinerated at high temperature in a dedicated factory). Healthcare associated waste is first heated and compressed, on the hospital site, and then assimilated to household waste. For household waste, incineration was performed corresponding to 374 kgCO_2_e per tons of waste of whom 214 kgCO_2_*e* were deducted for GHG emissions avoided due to energy production during incineration process. Household waste wasn’t separated into different material elimination processes as selective waste sorting isn’t performed yet.

Regarding medications, medicine manufactures were contacted, but no answer could be obtained or no data were available. Consequently, GHG emitted during manufacture of active ingredients, excipients and co-formulates wasn’t assessed because of the lack of reliable data. Parvatker et al. [8] conducted a process-based estimation of GHG emissions associated with the manufacturing of 20 anesthetic medicine, however few of them were analyzed in our study. In addition, the impact of solvents for injection drugs, like water for injection or saline solutions, which mostly remained on sterilization process, remains unknown.

### Economic input–output analysis

Because of the missing data on medications detailed above, and in order to more accurately estimate global warming impact of medications, an EIO analysis was performed on medicines, by using the monetary factor (MF) provided by the French Environment and Energy Management Agency (ADEME). For constituency, EIO analysis was also conducted on MD in a separated analysis.

MF is obtained through input–output tables, by adding an environmental layer. It allows to convert monetary flows into physical flows, such as GHG emissions, like kgCO_2_*e* [9], and is expressed in kgCO_2_*e* per euros (€). MF allows a more exhaustive estimation at a large scale, because it focuses on monetary values, which are more available than physical values. However, these large-scale calculations are also responsible for large variations in estimation [9].

MF used in our study, given by the ADEME, are 0.500 kgCO_2_*e*/€ for medicine and 0.315 kgCO_2_*e*/€ for MD and personal protective equipment.

### Energy and water consumption

Intensive Care Unit gas and electricity consumption was assessed. In our ICU, there are no specific meters. In order to estimate our annual electricity and gas consumption, the hospital's annual electricity and gas consumption (expressed in kWh) was indexed by the area of our intensive care unit. The indexed consumption was then multiplied by factors provided by the ADEME (0.0520 kgCO_2_*e*/kWh for electricity and 0.213 kgCO_2_*e*/kWh for gas for 2022 in France).

As laundry was situated in the hospital, energy (electricity and gas) of this infrastructure was taken into account with this estimation.

The same method was used for water consumption, using an emitting factor, providing by the ADEME of 0.394 kgCO_2_*e*/m^3^.

### Medical gas, caregivers transportation, alimentation and life support therapies

Medical gas consumption was analyzed through a hybrid-LCA. Noteworthy, only oxygen and nitrous oxide are used in our ICU, at the exception of sevorane as part of a dedicated randomized control trial. Our ICU does not have a dedicated gauge to accurately measure oxygen consumption. However, hospital oxygen consumption was obtained and indexed by ICU surface area to obtain an estimate of ICU oxygen consumption. GHG emissions related to O2 consumption were estimated through a hybrid LCA considering the emission factor of 0.0021 kgCO_2_*e*/L (or 2.1 kgCO_2_*e*/m3) retained by McGain and Seglenieks [10, 11]. Considering nitrous oxide, a factor of 273 kgCO_2_*e* per kg of gas was retained based on base empreinte recommendation. (See supplementary data for whole description of process base LCA methodology).

Hybrid LCAs were also performed for caregivers transportation, alimentation and life support therapies (Detailed method available in supplementary).

### Results presentation

Results are presented with the most reliable method considering each category. For MD, process-based LCA is reliable. Because of the lack of data, GHG emissions related to medications were presented through the EIO analysis. Other results (medical gas, energy, caregivers transportation, alimentation and life support therapies) are presented through hybrid-LCAs. Additional methods are available for comparison in supplementary.

### Hotspot identification

Hotspots with the highest global warming impact were identified as the 7 practices or products with the highest global warming impact among medicines and MD but also among other source of GHG emissions (energy, alimentation, transport). These hotspots highlight the areas where actions are most urgently needed.

### Objectives

The primary aim of the study was to identify hotspots of GHG emissions corresponding to individual activities or products responsible for the highest GHG emissions among MD and medicines used in our ICU. Secondary objectives were to estimate global GHG emissions of ICU activity, and respective GHG emissions of each studied category.

### Statistical analysis

Categorical variables were expressed as percentages and continuous variables as median and interquartile range. Mean consumption or GHG emissions per patient-days were obtain by dividing yearly consumption or GHG emissions by yearly number of patients-days.

## Results

### Setting

The ICU team was composed of 105 healthcare providers, with 40 caregivers working per day in average (14 nurses, 11 caregivers, 5 doctors, 5 residents, 3 administrative workers, 2 students). Common patient categories were sepsis, trauma, cardiological, renal and respiratory failure. Available organ replacement therapies were mechanical invasive and non-invasive ventilation, continuous renal replacement therapies, and extra corporeal membrane oxygenation (ECMO) support.

During 2022, 1, 021 patients were admitted to our ICU, representing 5, 820 patient-days. The median age was 66 years, the median Simplified Acute Physiology Score II (SAPSII) was 45. Two third (64%) were male, 63% were given invasive mechanical ventilation during their stay, representing 2, 182 ventilatory-days. 4 patients received extra corporal membrane oxygenation, representing 30 days of ECMO support, and there were 391 renal replacement therapy session. The overall mortality rate was 16.9%.

Forty-five MD references were analyzed (99% of MD consumption) while 101 medication references were studied (86% of medicines consumption).

### Green House Gas emissions by category

**Fig. 1:**
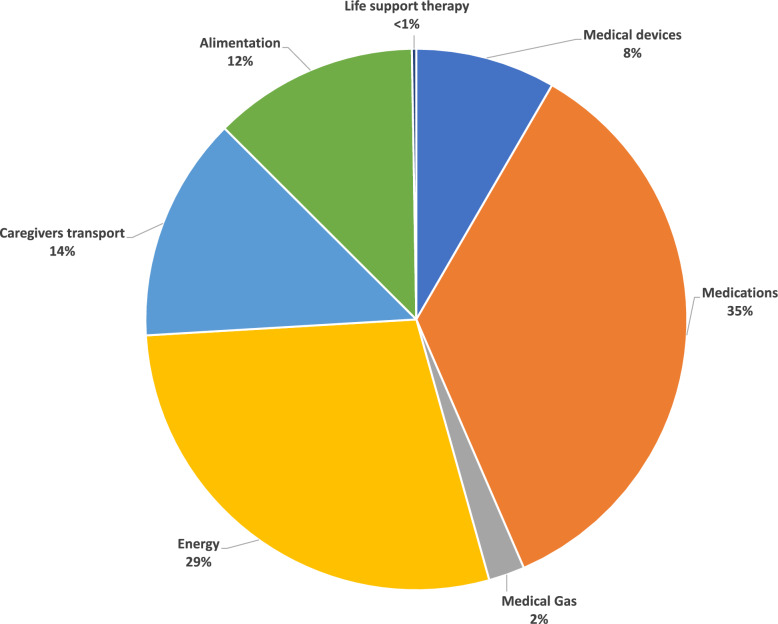
GHG emissions distribution by category (process based LCA for medical devices, EIO-analysis for medicine and hybrid LCA for other categories)

Green House Gas emissions were estimated at 61.1 kgCO_2_*e*/patient-day (process-based analysis for MD, and EIO analysis for medicine, hybrid LCA for other sources, cf supplementary data). Figure 1 reports the GHG emissions by category. Medications was considered as first category of emission (35%), followed by energy consumption (29%), caregivers transportation (14%), alimentation (12%) and then MD consumption (8%) (Fig. 1).

### Medications

**Fig. 2:**
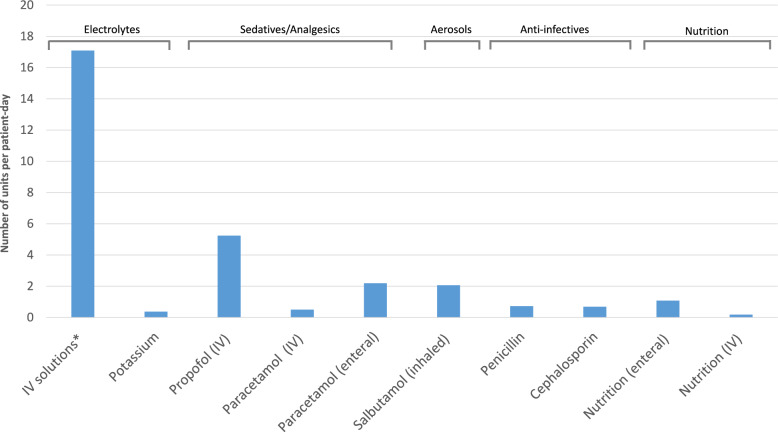
Consumption of the most used medications. Datas are expressed as units per patient-day. *”IV solutions” includes Water for injection (IV) (10–20 mL), Glucose 5% (IV) (10–1000 mL), and Sodium Chlorure 0,9% (IV) (10–1000 mL)

Forty-seven individual medicines were used per patient-day during study period. Most consumed medications were sterile water for injection (8.8 units per patient-day), propofol (5.3 units per patient-day), sodium chlorure (5.5 units per patient-day), Glucose 5% (2.7 units per patient-day) and paracetamol (2.2 units per patient-day) (see supplementary data for details regarding consumption of various presentation) (Fig. 2). Green House Gas emissions related to medications were estimated at 21.5 kgCO_2_*e* per patient-day. Importantly, injectable medications accounted for the most important part of GHG, estimated at 15.3 kgCO_2_*e* per patient-day (EIO analysis) (74% of GHG emissions related to medications).

### Energy and water consumption

During 2022, 113.5 kWh per patient-day were consumed, consisting of 71.2 kWh of gas and 42.4 kWh of electricity consumption; corresponding to 17.4 kgCO_2_*e* per patient-day (15.2 kgCO_2_*e* per patient-day for gas and 2.2 kgCO_2_*e* per patient-day for electricity).

320 litters of water (or 0.32m^3^) per patient-day were consumed, representing 0.1 kgCO_2_*e* per patient-day.

### Professional transport and meals

Professional alimentation during duty accounted for 14, 600 meals per year, corresponding to an estimation of 29, 930 kgCO_2_*e* per year, representing 5.2 kgCO_2_*e* per patient-day. In the same time, 6, 629 meals were delivered to ICU patients, corresponding to an estimation of 13, 589 kgCO_2_*e* per year, representing 2.3 kgCO_2_*e* per patient-day.

Among the 47 professionals who responded to transport survey, average home-to-work distance was 14 km (median) [6–25] (interquartile range). Private car was dominant (83% of travels) with about 50% of the caregivers reporting carpooling with a median frequency of 1 travel over 7. Thirty-four caregivers (72%) reported using occasionally or regularly a sustainable mode of transport.

Based on the answers, GHG emissions related to professional transport were estimated at 8.2 kgCO_2_*e* per patient-day with private car being responsible for 92% of GHG emissions (7.5 kgCO_2_*e* per patient-day).

### Medical devices

**Fig. 3:**
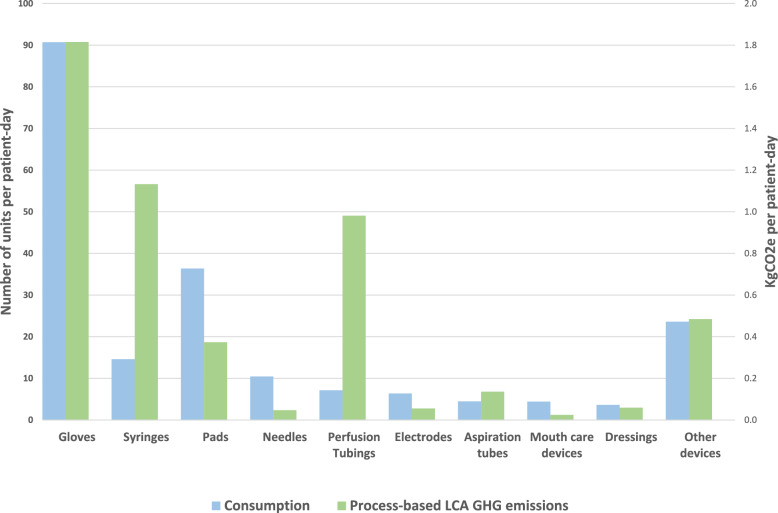
Consumption and GHG emissions of the most used medical devices. Datas are expressed as units per patient-day for consumption, and as kgCO2e per patient-day for GHG emissions

In 2022, 202 individual MD were used per patient-day, equivalent to 5.1 kgCO_2_*e* per patient-day. Figure 3 depicts the 10 most consumed MD and their related GHG emissions according to process-based analysis.

Gloves accounted for the main part of the impact (representing 1.8 kgCO_2_*e* per patient-day).

Devices used for intravenous administration accounted for only 16% of the total consumption of MD, but represented 42% of GHG emissions related to all MD. Specifically, syringes (1.1 kgCO_2_*e* per patient-day), perfusion tubings (0.9 kgCO_2_*e* per patient-day) and gauze pads (0.4 kgCO_2_*e* per patient-day) were the 3 most important sources of MD related GHG emissions after gloves.

### Medical gas consumption

Overall, 3, 132, 639 L of oxygen and 12 bottles of 1.5 m^3^ of equimolar mix of nitrous oxide/oxygen were consumed during study period. Green House Gas emissions related to oxygen consumption were estimated at 1.1 kgCO_2_*e* per patient-day and those of nitrous oxide were estimated at 0.2 kgCO_2_*e* per patient-day.

### Life support therapies

Extra Corporeal Membrane Oxygenation and renal replacement therapy were considered to be responsible for the emission of 0.02 and 0.1 kgCO_2_*e* per patient-day respectively.

### Waste

We estimated that 6.3 tons of hazardous waste were eliminated in 2022, while household waste was estimated at 3.6 tons. Considering the 24.5 km between the incineration site (Planguenoual, Côtes d’Armor, Bretagne, France) and the hospital, GHG emitted for waste transportation was estimated at 33 kgCO_2_*e* for the entire year (less than 0.1 kgCO_2_*e* per patient days). GHG emitted during waste incineration was included in GHG emissions calculation of each MD and medicines.

## Discussion

We conducted an exhaustive evaluation of GHG emissions related to MD and medicines used in an ICU during an entire year and identified main sources of GHG emissions.

We identified 7 hot spots of GHG emissions responsible for more than 80% of whole GHG emissions of our ICU. Injectable medications were the main source of GHG emissions, based on EIO analysis. Gas consumption was considered as the second hot spot. Caregivers transportation (especially private car) and alimentation were the third and fourth hot spots. Regarding MD, gloves were the most emissive device identified, followed by syringes and perfusion tubings.

Our results are partially consistent with previous calculations made by different teams and studies. In 3 Australian and American studies [5, 6], McGain and Prasad estimated GHG emissions in between 88 and 178 kgCO_2_*e*/patient/day, higher than the 61 kgCO_2_*e*/patient/day estimated in our ICU. Differences can be explained by disparities in energy consumption and production. First, consumption was higher in their studies. Then, source of electricity production differs. In France, most of the energy is produced from nuclear and hydraulic technologies with a minimal contribution from fossil fuel combustion (less than 16%), while it is mainly produced with fossil fuel combustion in the USA and Australia (60–68%) [12, 13]. Importantly, energy consumption in our ICU was no directly measured but only estimated, and so GHG emissions might be higher than reported. Even though, energy would still be one of the first factor of emissions in our specific ICU. Interestingly, when only consumables and waste were considered, remaining GHG emissions were estimated in between 20 and 25 kgCO_2_*e*/patient/day in the American and Australian study respectively [5, 6], as compared with 26 kgCO_2_*e*/patient/day in the present work.

Transport is also an important factor. In our rural region, public transport isn't well developed. Sustainable mobility, such as carpooling, is crucial. However, this is highly dependent on the location of hospitals. Furthermore, cycling has a benefic impact, non-only on GHG emissions, but also on health, as it helps to prevent chronic diseases and decreases medical and personal costs [14].

Regarding medications, despite attempts to contact pharmaceutical manufacturers, no data could be obtained. Data must be shared and fully available in order to better guide healthcare givers when a medication has to be evaluated. However, in both models (process based and EIO), injectable medications were responsible for the higher part of GHG (Supplementary data). We identified 2 actions to reduce medications impact. The first is to prioritize enteral administration when possible. In the case of medicines such as paracetamol, this not only reduces the carbon footprint but can also benefit patients by reducing hypotension incidence [15–17]. The second step is to optimize intravenous administration. Allowing higher concentrations, such as switching from 1 to 2% Propofol, can save up to 1, 396 kgCO_2_*e*/year (including diminution of GHG emissions through reduction of syringe consumption). Similarly, anti-infectious administration can be optimized [18].

Regarding MD, our results differed slightly with those reported by Hunfeld et al. [7], where MD and some medicine packages represented 12 kgCO_2_*e*/patient/day. The difference can be explained by a lower number of MD used in our ICU and by differences in footprint calculation approaches. Hunfeld et al. performed a material flow analysis focusing on the inputs and outputs of the ICU, whereas we chose an LCA based approach with a closer look at each device, allowing us to detail specific GHG emissions. Regarding personal protective equipment, gloves were identified as the major contributor of GHG emissions. Some studies questioned the use of gloves during care, and highlighted their misusage and their overuse. Rigorous use of gloves can improve both quality of care and GHG emissions [19–21]. Interestingly, through campaigns such as Great Osmond Street Hospitals' "The gloves are off" campaign [22], we expect to reduce glove consumption by 30%, saving 2, 638 kgCO_2_*e*/year. Importantly, focusing on highly consumed items such gloves seems more effective than focusing on more complex but less used items. As an example, whole bronchoscopy activity during 2022 in our ICU was estimated at 298 kgCO_2_*e*/year only, which represents less than 4% of GHG emissions related to gloves [23]. Similarly, ECMO and renal replacement therapy were only responsible for marginal GHG emissions of our ICU during 2022. Hunfeld et al. already highlighted that this life supports constituted a relatively small part of material flow in their ICU [7].

Other way of reducing MD related GHG emissions may resides in the development of reusable items. Several studies already highlighted this solution, especially for intensive care specific items like laryngoscopes or bronchoscopes [23–25].

Our study has several limitations. First, these calculations have been conducted by 2 healthcare providers and not by eco-audit professionals. However, it allows to project into concrete and realizable actions. Second, despite our ICU activity being similar with those of most French ICUs [26], some data like energy consumption and professional transport cannot be transposed in other places, as it mainly depends on local characteristics, which limits external validity of our results. Third, we used GHG emissions factors given by the ADEME, which gives a reliable order of magnitude but not the real GHG emissions. More studies are needed to calculate and refine GHG emissions factors in order to obtain more accurate GHG emissions calculations.

## Conclusion

Main hot spots of GHG emissions directly related to critical care were injectable medications, gloves, syringes and perfusion tubings. There is still a lack of data on the GHG emissions related to medicine production, so it is very likely that these results will evolve as these values become available. Interestingly, gas consumption, professional transportation and alimentation during duty were also major sources of GHG emissions. Upcoming studies focusing on actions on these particular hot spots would be of interest in order to significantly decrease GHG emissions but also to increase resilience of critical care.

## Supplementary Information

Supplementary material 1.

Supplementary material 2.

## Data Availability

The datasets generated during the current study are available from the corresponding author on reasonable request.
