# Supplementary material for: Which carbon footprint for my ICU? Benchmark, hot spots and perspectives
Source: Ann Intensive Care. 2025 Mar 20;15:35. doi: 10.1186/s13613-025-01445-z (PMC11925816; doi:10.1186/s13613-025-01445-z)
Supplement: Supplementary file 1 — Supplementary material 1. [file 13613_2025_1445_MOESM1_ESM.docx]

***Inflows and outflows***

Inflow datas for the year 2022 were provided by the purchasing department of the hospital, which exhaustively covers the range of medicines, medical devices and personal protective equipment (PPE) given to each unit in the hospital.

Outflows correspond to waste and unused parts of products. The number of units used is the same as for inflows, as our ICU operates with very low material reserves.

***Transportation and Meals.***

A survey was sent to the 105 healthcare providers in our ICU and 47 (44.7%) responses were received. The main and secondary modes of transport and the distance from home to work were assessed.

Analyses were performed for each occupation category, allowing an exhaustive estimation by multiplying these results by the number of healthcare providers of each category shifted each day.

In Saint-Brieuc hospital, meals for caregivers are provided at two hospital cafeterias that are supplied by the central hospital kitchen, localized inside the hospital building. Cooks and kitchen team are involved in the “comité de dévellopement durable” to improve hospital sustainability. We assume that actions conducted by this committee may reduce GHG related to alimentation of the staff. Consequently, it seems important to include this source of GHG emissions as part of our research. The impact of patients and caregivers meals was assessed by multiplying the annual number of meals consumed during working hours by a standard meal factor impact given by the ADEME (2.05 kgCO_2_*e* /meal).

***Life support therapy***

For ECMO and renal replacement therapy, devices and consumables were both analyzed. Composition of membrane was assimilated to polyurethane (emission factor of 4.37 kg CO_2_*e*/kg). GHG related to devices use was estimated using a hybrid LCA considering the industrial machines emission factor of 5.5 kgCO_2_*e* per kg of machines. GHG related to devices were considered to be equally divided into the 10 years of expected function.

***Cleaning devices***

Products use to clean surfaces were not assessed as there were inconsistency in the data provided by the cleaning team (external activity).

***Waste***

Weight of waste was approximated considering the weight of all MD, related packaging but also the weight of pharmaceutical packaging. Waste transport related GHG emission was estimated using the emission factor for transport by truck (0.0799 kgCO_2_*e*/ton/km for French transportation) considering the 24 km between the hospital and the incineration factory.

Noteworthy, biological fluids were evacuated through a dedicated water network but volume could not be quantified. Consequently, GHG related to elimination of biological effluent (urine and feces) could not be included in the present analysis. Importantly, GHG emitted during waste incineration was included in GHG emissions calculation of each MD and medicines.
